# Combining the GP’s assessment and the PHQ-9 questionnaire leads to more reliable and clinically relevant diagnoses in primary care

**DOI:** 10.1371/journal.pone.0276534

**Published:** 2022-10-21

**Authors:** Clara Teusen, Alexander Hapfelmeier, Victoria von Schrottenberg, Feyza Gökce, Gabriele Pitschel-Walz, Peter Henningsen, Jochen Gensichen, Antonius Schneider, for the POKAL-Study-Group

**Affiliations:** 1 Institute of General Practice and Health Services Research, TUM School of Medicine, Technical University of Munich, Munich, Bavaria, Germany; 2 Institute for AI and Informatics in Medicine, TUM School of Medicine, Technical University of Munich, Munich, Bavaria, Germany; 3 Dept. of Psychosomatic Medicine and Psychotherapy, University Hospital TU Munich, Munich, Bavaria, Germany; 4 Institute of General Practice and Family Medicine, University Hospital of the Ludwig-Maximilians-University of Munich, Munich, Bavaria, Germany; Universidade Federal do Rio Grande do Sul, BRAZIL

## Abstract

**Background:**

Screening questionnaires are not sufficient to improve diagnostic quality of depression in primary care. The additional consideration of the general practitioner’s (GP’s) assessment could improve the accuracy of depression diagnosis. The aim of this study was to examine whether the GP rating supports a reliable depression diagnosis indicated by the PHQ-9 over a period of three months.

**Methods:**

We performed a secondary data analysis from a previous study. PHQ-9 scores of primary care patients were collected at the time of recruitment (t1) and during a follow-up 3 months later (t2). At t1 GPs independently made a subjective assessment whether they considered the patient depressive (yes/no). Two corresponding groups with concordant and discordant PHQ-9 and GP ratings at t1 were defined. Reliability of the PHQ-9 results at t1 and t2 was assessed within these groups and within the entire sample by Cohen’s Kappa, Pearson’s correlation coefficient and Bland-Altman plots.

**Results:**

364 consecutive patients from 12 practices in the region of Upper Bavaria/Germany participated in this longitudinal study. 279 patients (76.6%) sent back the questionnaire at t2. Concordance of GP rating and PHQ-9 at t1 led to higher replicability of PHQ-9 results between t1 and t2. The reliability of PHQ-9 was higher in the concordant subgroup (κ = 0.507) compared to the discordant subgroup (κ = 0.211) (p = 0.064). The Bland-Altman Plot showed that the deviation of PHQ-9 scores at t1 and t2 decreased by about 15% in the concordant subgroup. Pearson’s correlation coefficient between PHQ-9 scores at t1 and t2 increased significantly if the GP rating was concordant with the PHQ-9 at t1 (r = 0.671) compared to the discordant subgroup (r = 0.462) (p = 0.044).

**Conclusions:**

The combination of PHQ-9 and GP rating might improve diagnostic decision making regarding depression in general practices. PHQ-9 positive results might be more reliable and accurate, when a concordant GP rating is considered.

## Introduction

Epidemiological studies show that depression is one of the major health problems worldwide [1, 2]. Thus, the accurate diagnosis and appropriate treatment of patients with depressive disorder is a key challenge to our health system [3–5]. Often, the first point of contact for patients with depression is the general practitioner (GP) [6]. Differentiating depressive symptoms from somatic, non-specific, functional or somatoform body complaints is difficult for GPs [7], particularly if patients are multimorbid and seek help for physical rather than psychological complaints [8–10]. Furthermore, compared to specialists in mental health care, the work of GPs takes place in a setting with a high risk of both over and under diagnosis of depression due to the presence of multimorbidity [3]. Even though about 10% of primary care patients are likely to meet criteria for major depression, detection and treatment rates are still low [11]. In addition to that, the low-threshold access to patients in general practices and the confrontation with subthreshold symptoms that could indicate a multitude of possible diseases complicate the correct diagnosis of depression [12, 13]. However, a correct diagnosis is essential for further adequate treatment of depression.

Previous studies investigated how GPs deal with the challenges of diagnostic decision making regarding depression. It was shown that the GP’s approach often differs from common psychiatric diagnostic systems like the International Statistical Classification of Diseases and Related Health Conditions (ICD-10) or the Diagnostic and Statistical Manual of Mental Disorders (DSM-V) [14]. According to ICD-10 and DSM-V, a depressive disorder can be diagnosed after a 2-week reference period [15]. In general practice, this time criterion is sometimes considered too short to confirm a depression diagnosis [16]. Usually, only if depressive symptoms persist for a longer period of time a depression diagnosis is considered and discussed carefully with the patient. Accordingly, GPs may be better at diagnosing correctly more severe than mild depression [17], in part because this subtype of depression is associated with a longer duration of symptoms [18]. Other studies found that the 2-week reference period for depression is appropriate and valid in general practice [19, 20]. However, in order to initiate even better diagnosis for depression in primary care, it might be helpful to take into account GP heuristics, their diagnostic strategies and thought processes in addition to existing psychiatric diagnostic criteria [3, 21–24]. A systematic review identified the "experienced anamnesis" and the patient’s long-term history as decisive factors for the GP’s diagnosis [25]. The length of the doctor-patient relationship is thus an important factor for GP diagnostics [26]. Besides these, other heuristics that GPs use for diagnostic decision making are watchful waiting, consideration of etiological and contextual factors and stepwise diagnostic procedures [25, 27].

A common strategy to improve diagnostic decision making is the introduction of screening questionnaires [28]. It has been shown that the systematic use of validated screening tools can improve detection and diagnosis of depression in primary care [29]. However, the use of depression screening questionnaires in primary care is discussed controversially, as a screening strategy leads to an over-estimation of the depression prevalence [30]. For example, screening questionnaires like the Patient Health Questionnaire 9 (PHQ-9) lead to high false-positive rates due to the low pre-test probability of depression in primary care [30]. In addition, the diagnosis of depression is affected by the correlation between somatic illnesses and the somatic symptoms of depression [31, 32]. Whereas a PHQ-9 positive result with a PHQ-9 score ≥10 may represent clinically significant depressive symptoms, a structured diagnostic interview is needed to confirm the presence of major depressive disorder. Beyond that, the PHQ-9 seems to be more suitable for ruling-out than for ruling-in the diagnosis of depression in primary care [33–35]. Therefore, there are conflicting opinions about the recommendation of routinely screening for depression in primary care [36]. The Canadian Task Force on Preventive Health Care [37] and the guideline for depression management from the United Kingdom’s National Institute for Health and Clinical Excellence [34] do not recommend routinely screening for depression in primary care settings whereas the US Preventive Services Task Force recommends a universal screening approach for depression in the general adult population [38]. We suppose that a simplified combination of screening questionnaires and GP heuristics could be useful to improve diagnostic quality and practicability in primary care.

The aim of the present analysis is to examine whether the GP rating of the patient as depressive is related to the persistence of depressive symptoms indicated by the PHQ-9 at the time of recruitment and during a follow up three months later. We assume that a longer duration of symptoms as determined with repeated PHQ-9 measurements is stronger associated with a depression diagnosed by the GP.

## Methods

### Study design and sample

We performed a secondary data analysis from a previous study on the impact of a complex educational intervention on diagnostic accuracy of impaired mental health in general practices [26]. 12 general practitioners in the region of Upper Bavaria/Germany agreed to take part in this longitudinal study. Suitable practices were recruited through the network of the 210 general practitioners with teaching duties at the Technical University of Munich (TUM). The data collection was carried out between March and October 2014.

All consecutive adult patients who attended the practices in the study period on certain days at regular intervals were asked in the waiting room by a research assistant to fill in a PHQ-9 questionnaire before seeing the doctor (t1). Additionally, at time point t1, the GPs made a subjective assessment whether they considered the patient depressive (yes/no). GPs were blinded to the PHQ-9 result of the patients they were supposed to assess for depression. During a follow-up investigation three months later (t2), patients were invited to fill in the PHQ-9 again to investigate the stability of the depressive symptoms. The questionnaire was sent by post and patients were asked to return it to the Institute of General Practice and Health Services Research. The patients received an incentive of 10 € per completed questionnaire. Inclusion criteria were an age of at least 18 years, sufficient knowledge of the German language and a signed consent form. Patients were not asked if they received any therapy, counselling or medication in the meantime. The underlying data for this study are pseudonymized and the study was approved by the Medical Ethics Committee of the Technical University of Munich (approval No 15/14).

### Questionnaire

The validated German version of the Patient Health Questionnaire 9 (PHQ-9) was used as a screening questionnaire to assess the presence of depressive symptoms in the patients within the past two weeks [39]. The depression severity score comprises nine items which can be summarized, with a range from zero (no depression) to 27 (maximum). Findings from previous studies show that the use of a cut-off value of 10 or higher is considered useful [40] as a score of 10 represents at least a moderate level of depressive symptoms [39]. A score between five and 10 is mostly found in patients with mild or subthreshold depressive symptoms and corresponds to a mild degree of severity [39]. Further on, the questionnaire presented to the patients at t1 comprised sociodemographic items regarding education, occupation and family status.

### Data analysis

The distribution of quantitative data is described by mean values and standard deviations. Qualitative data is presented by absolute and relative frequencies. Statistical significance of respective group differences was assessed by Chi-Squared Tests and t-Tests. The main research question was whether patients’ self-ratings on the PHQ-9 at t1 and t2 were more reliable when the GP’s rating matched with the PHQ-9 assessment at t1, compared to when there was no match. For this purpose, a PHQ-9 ≥10 was used to indicate a self-rated depression. This outcome was labelled as PHQ-9 positive, PHQ-9 <10 was labelled as PHQ-9 negative. Two groups with concordant versus discordant PHQ-9 and GP ratings at t1 were defined. The replicability of PHQ-9 results between t1 and t2 was compared between the concordant and discordant group by a 2 x 2 table. Mean values were presented additionally to enable a comparison of the dimensional PHQ-9 results. Furthermore, reliability of the PHQ-9 test results at t1 and t2 was assessed within these groups and within the entire sample by Cohen’s Kappa, Pearson’s correlation coefficient and Bland-Altman plots. Limits of Agreement (LoA) which cover about 95% of the differences between measurements were computed for the latter. Respective hypothesis testing of group differences was performed by Z-tests. All analyses were performed using the software package SPSS (Version 25, IBM, Armonk, NY, USA) and R 4.0.3 (The R Foundation for Statistical Computing, Vienna, Austria). Exploratory two-sided 5% significance levels were used for any hypothesis testing.

Within the previous cluster randomised controlled pilot study the 12 practices were divided into a control and an intervention group after a one-time training intervention for general practitioners [26]. The GPs in the intervention group received a one-day training on diagnostics and interviewing as well as on recognising and dealing with psychosomatic patients. The training included expert lectures (on depression, anxiety and somatization), group discussions and acting out psychosomatic counselling situations with an acting patient. However, a one-day training intervention alone did not seem to improve the perception and management of psychosomatic illness [26]. No relevant sociodemographic or diagnostic differences were found between the intervention and the control group, so that collected data of all patients were merged in our secondary analysis.

## Results

**Fig 1:**
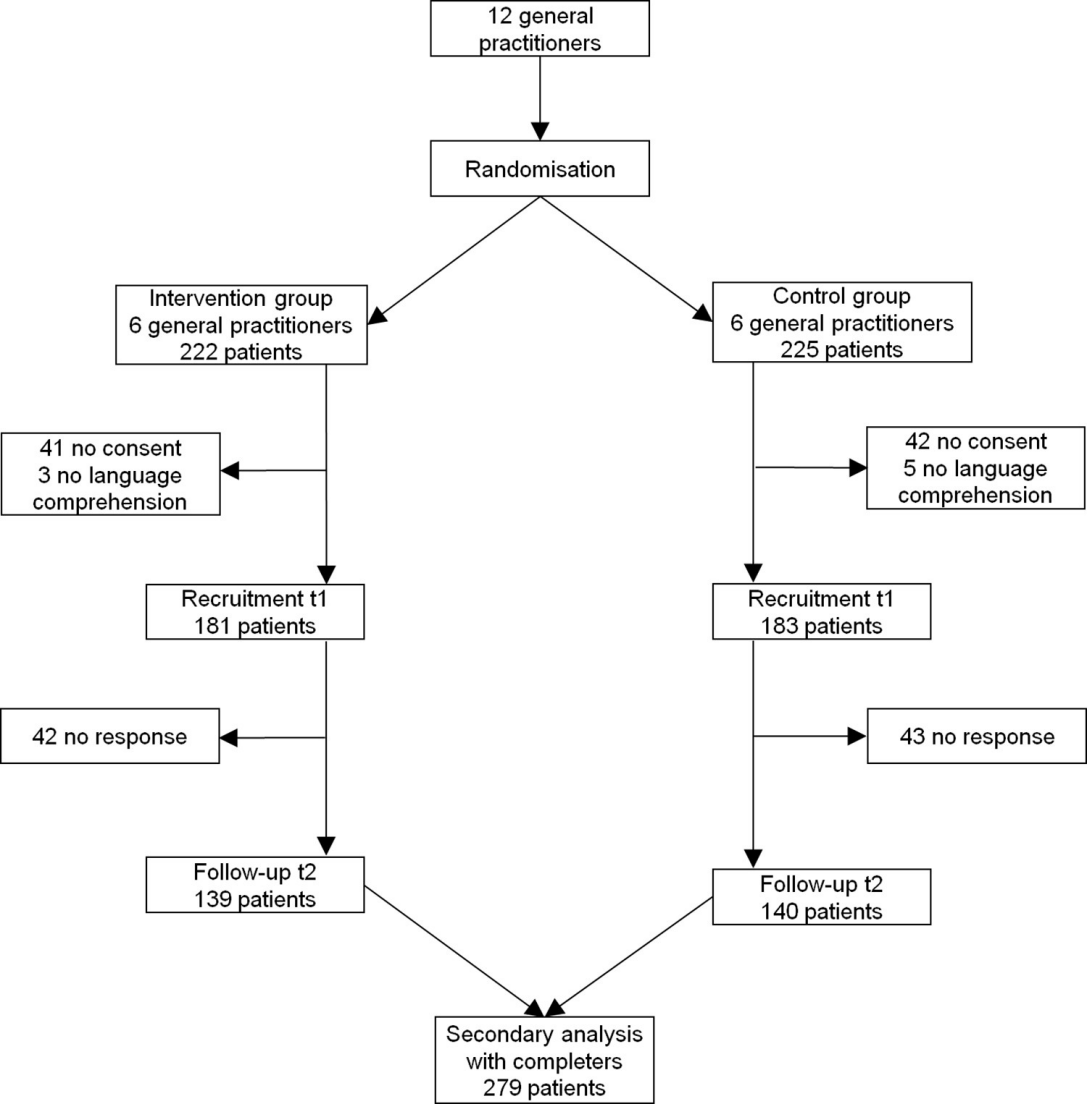
Flowchart of patients.

364 consecutive patients from 12 practices in the region of Upper Bavaria/Germany participated in this longitudinal study (see Fig 1). 279 patients (76.6%) sent back the questionnaire at t2. Non-responder analysis showed no relevant differences with respect to gender and depression diagnosis by the GP or PHQ-9 (not in Table 1). However, the average age of non-responders (mean 47.7, standard deviation 19.0) was significantly (p = 0.043) lower compared to responders (mean 52.3, standard deviation 18.1). The baseline characteristics of the patients at t1 are displayed in Table 1.

**Table 1:** Baseline characteristics (t1).

		Depression rating by the GP^1^			Depression at t1 (PHQ-9 ≥10)^2^		
**Parameter (Missing values)**	Total (n = 364) n(%)	Yes (n = 85) n(%)	No (n = 271) n(%)	P-Value	Yes (n = 61) n(%)	No (n = 290) n(%)	P-Value
Sex, female (0)	203 (55.8)	55 (64.7)	142 (52.4)	p = 0.046	38 (62.3)	159 (54.8)	p = 0.285
Age (6) [Mean (SD)] in years	51.24 (18.41)	50.98 (18.56)	51.16 (18.23)	p = 0.803	45.82 (16.95)	51.90 (18.61)	p = 0.158
Marital status (2)				p = 0.087			p<0.001
Married or in a stable relationship	245 (67.3)	50 (58.8)	193 (71.2)		27 (44.3)	210 (72.4)	
Single	92 (25.3)	29 (34.1)	61 (11.4)		27 (44.3)	60 (20.7)	
Widowed	25 (6.9)	6 (7.1)	17 (6.3)		6 (9.8)	19 (6.6)	
Education level (2)				p = 0.485			p = 0.659
No school degree	6 (1.6)	3 (3.5)	3 (1.1)		2 (3.3)	4 (1.4)	
< 10 y of formal education	107 (29.4)	26 (30.6)	79 (29.2)		15 (24.6)	83 (28.6)	
10 y of formal education	112 (30.8)	28 (32.9)	81 (29.9)		22 (36.1)	88 (30.3)	
12–13 y of formal education	116 (31.9)	23 (27.1)	92 (33.9)		17 (27.9)	97 (33.3)	
Other	21 (5.8)	5 (5.9)	15 (5.5)		4 (6.6)	17 (5.9)	
Occupation (5)				p = 0.017			p<0.001
Employed part-time	61 (16.8)	17 (20.0)	43 (15.9)		12 (19.7)	46 (15.9)	
Employed full-time	146 (40.1)	26 (30.6)	119 (43.9)		17 (27.9)	126 (43.4)	
Housewife/homemaker/non-working	16 (4.4)	3 (3.5)	13 (4.8)		5 (8.2)	11 (3.8)	
Retired	94 (25.8)	23 (27.1)	68 (25.1)		10 (16.4)	80 (27.6)	
Unemployed	10 (2.7)	7 (8.2)	3 (1.1)		7 (11.5)	3 (1.0)	
Other	32 (8.8)	7 (8.2)	23 (8.5)		8 (13.1)	22 (7.6)	

^1^ 8 missings due to missing GP rating

^2^ 13 missings due to incomplete PHQ-9 response. SD: Standard deviation.

Table 2 depicts the replicability of the PHQ-9 results over the period from t1 to t2. Related to the entire sample, 47 patients (17.9%) were PHQ-9 positive at t1; and 216 patients (82.1%) were PHQ-9 negative. 25 (53.2%) of the PHQ-9 positives received a positive result at t2 again. 194 patients (89.8%) without depression at t1 remained without depression at t2.

**Table 2:** Replicability of the PHQ-9 results at t1 and t2 alone compared to the stability of the PHQ-9 results in the PHQ-9 and GP rating concordant vs. discordant subgroup at t1.

	All responders^1^ (N = 263)						
	Depression (PHQ-9≥10) at t2	PHQ-9 at t1	PHQ-9 at t2	No depression (PHQ-9<10) at t2	PHQ-9 at t1	PHQ-9 at t2	All
Depression (PHQ-9≥10) at t1	25 (53.2)	15.2±4.9	15.2±3.5	22 (46.8)	12.4±2.1	6.1±1.9	47 (17.9)
		14; 10–27	15; 10–25		12; 10–17	6.7; 1–9	
No depression (PHQ-9<10) at t1	22 (10.2)	5.3±2.9	12.2±2.4	194 (89.8)	3.1±2.5	3.3±2.2	216 (82.1)
		5; 0–9	11; 10–18		2.5; 0–9	3; 0–9	
		**Concordant subgroup at t1 (N = 208)**					
	Depression (PHQ-9≥10) at t2	PHQ-9 at t1	PHQ-9 at t2	No depression (PHQ-9<10) at t2	PHQ-9 at t1	PHQ-9 at t2	All
Depression (PHQ-9≥10) at t1	17 (63.0)	16.6±5.3	15.5±3.9	10 (37.0)	12.3±2.3	6.2±1.4	27 (13.0)
		17; 10–27	15; 10–25		12; 10–16	6.4; 4–8	
No depression (PHQ-9<10) at t1	15 (8.3)	4.7±2.9	11.8±2.2	166 (91.7)	2.9±2.4	3.1±2.1	181 (87.0)
		4; 0–9	11; 10–16		2; 0–9	3; 0–9	
		**Discordant subgroup at t1 (N = 55)**					
	Depression (PHQ-9≥10) at t2	PHQ-9 at t1	PHQ-9 at t2	No depression (PHQ-9<10) at t2	PHQ-9 at t1	PHQ-9 mean t2	All
Depression (PHQ-9≥10) at t1	8 (40.0)	12.1±2.0	14.7±2.8	12 (60.0)	12.5±2.0	6.0±2.4	20 (36.4)
		12; 10–16	14; 12–20		12; 10–17	6.9; 1–9	
No depression (PHQ-9<10) at t1	7 (20.0)	6.7±2.1	13.1±2.8	28 (80.0)	4.3±2.8	4.3±2.5	35 (63.6)
		7; 4–9	13; 10–18		3.5; 0–9	4; 0–9	

^1^With complete PHQ-9 response; Descriptive statistics are n (%), Mean ± Standard Deviation and Median (Range).

The PHQ-9 test result in terms of inclusion or exclusion of depression was concordant with the GP rating at t1 in 208 patients. In this concordant subgroup, 27 patients (13.0%) received a PHQ-9 positive result at t1. 17 of these patients (63.0%) were PHQ-9 positive at t2 again. 181 patients (87.0%) were PHQ-9 negative at t1; and 166 patients (91.7%) were PHQ-9 negative at t2 again.

The discordant group with a mismatch between PHQ-9 and GP assessment at t1 comprised 55 patients. In this subgroup, 20 patients (36.4%) were PHQ-9 positive at t1. 8 of these patients (40.0%) received this positive result at t2 again. 35 patients (63.6%) were PHQ-9 negative at t1; and 28 patients (80.0%) were PHQ-9 negative at t2 again. The PHQ-9 mean values were higher in the concordant subgroup compared to the discordant subgroup in case of PHQ-9 positives at t1. Likewise, the PHQ-9 mean values were lower in the concordant subgroup compared to the discordant subgroup in case of PHQ-9 negatives at t1, suggesting that PHQ-9 means are more clearly positioned above or below the cut-off value (≥10) in the concordant subgroup.

The PHQ-9 results at t1 and t2 showed a moderate agreement overall (κ = 0.430, SE = 0.072). They were higher if the GP rating was concordant with the PHQ-9 test result at t1 (κ = 0.507, SE = 0.086) compared to a fair agreement in the discordant group (κ = 0.211, SE = 0.135), still this comparison was not significant (p = 0.064) (data not in Table 2).

**Fig 2:**
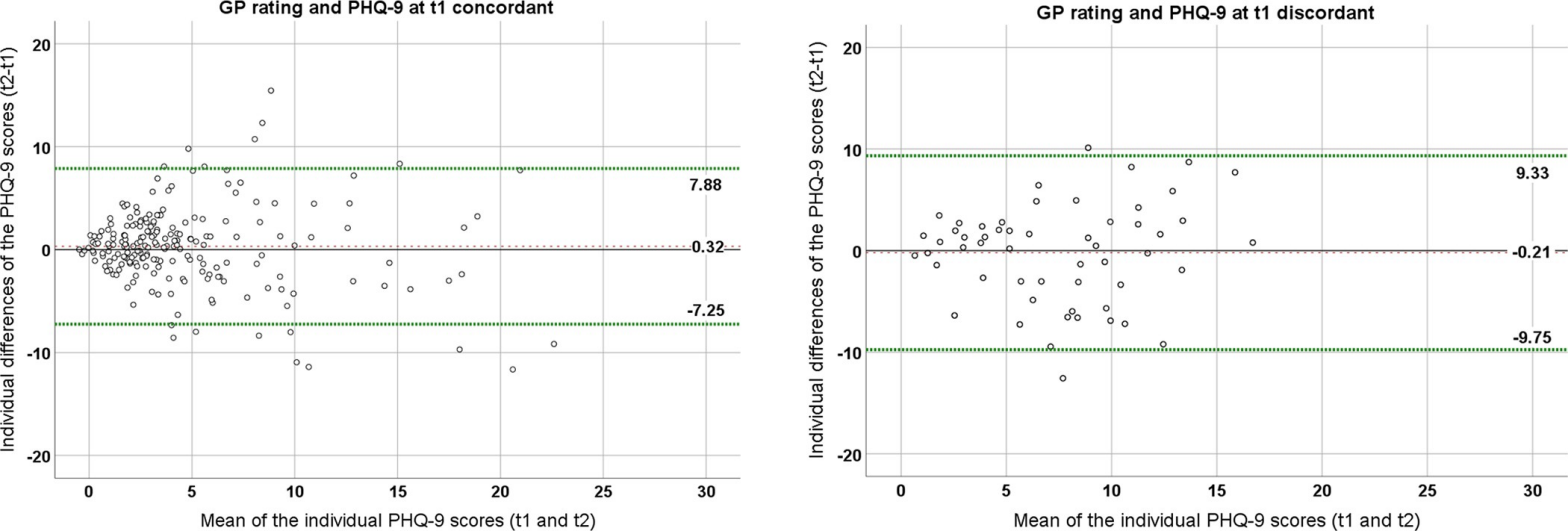
Bland-Altman Plots of the subgroups with a concordant vs. discordant GP rating and PHQ-9 results at t1 (dashed lines represent the Limits of Agreement and the bias).

The Bland-Altman Plot (Fig 2) indicates that the agreement of PHQ-9 scores at t1 and t2 for the concordant subgroup is higher (Limits of Agreement (LoA): -7.25 to 7.88) than in the discordant subgroup (LoA: -9.75 to 9.33). The LoA are 95% prediction intervals describing the range in which the majority of the individual differences between the PHQ-9 measurements at t1 and t2 are expected to lie, as they cover about 95% of these values. The deviation of PHQ-9 scores at t1 and t2 decreases by about 15% when the GP rating is concordant with the PHQ-9 at t1. The plots show that patients with a PHQ-9 mean between 5 and 10 had large absolute differences between t1 and t2, suggesting high variability of the PHQ-9 scores in this range. Patients with a PHQ-9 mean <5 and a PHQ-9 mean ≥10 showed lower absolute differences between the time points, especially in the concordant subgroup.

**Fig 3:**
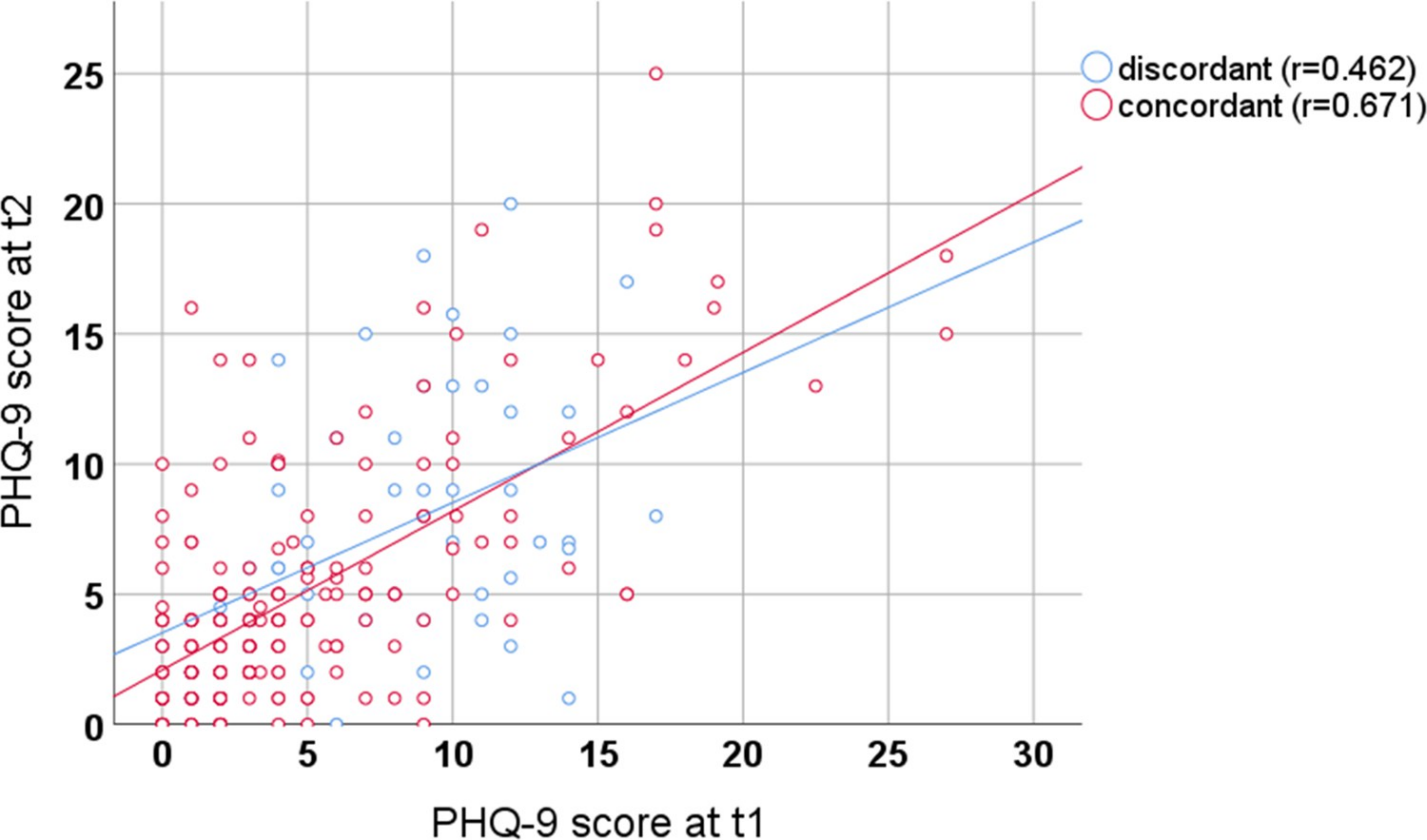
Correlation of PHQ-9 scores at t1 and t2 with discordant vs. concordant PHQ-9 and GP rating.

A scatter plot regarding the correlation of PHQ-9 scores at t1 and t2 is given in Fig 3. Pearson’s correlation coefficient between PHQ-9 scores at t1 and t2 was r = 0.646. Pearson’s correlation coefficient increased significantly if the GP rating was concordant with the PHQ-9 at t1 (r = 0.671) compared to the discordant subgroup (r = 0.462) (p = 0.044). All correlation coefficients were statistically significant (p<0.001).

## Discussion

The analysis showed that the concordance of the GP rating and the PHQ-9 results at t1 leads to a higher replicability of a PHQ-9 positive result over a period of three months. In addition, the replicability of a PHQ-9 negative result was improved, when GP rating and PHQ-9 were concordant at t1.

We found for the entire sample, that only 53.2% of all PHQ-9 positives at t1 received the same PHQ-9 positive result at t2. This proportion increased to 63.0% if the GP rating and PHQ-9 results were concordant at t1.

Beyond that, the Bland-Altman Plot showed a higher agreement of PHQ-9 scores between baseline and follow-up in the concordant subgroup. An increased reliability was also indicated by the significantly higher correlation of PHQ-9 scores between t1 and t2 in the concordant subgroup. Assuming that the persistence of symptoms is more strongly associated with actual depression [17, 18], our results suggest that a better ruling-in of depression is achieved when the GP rating in addition to a high PHQ-9 score is considered. The explicit consideration of GP heuristics like the "experienced anamnesis", the patient’s long-term history [25], watchful waiting, consideration of etiological and contextual factors and stepwise diagnostic procedures [25, 27] could be of great use to increase the pre-test probability of depression in primary care. Therefore, as the mere use of screening questionnaires in general practices does not lead to sufficient diagnostic certainty [30], the combination of PHQ-9 and the GP rating might improve the detection of patients with depression in primary care.

Studies have shown that the false-positive rate of the PHQ-9 is around 60% in a population with a 10% prevalence of depression so that false-positives and false-negatives have to be examined carefully [30]. Overall we found that 46.7% of the patients with a PHQ-9 positive result at t1 had a negative result at t2 in the present study. This may indicate a significant overestimation of PHQ-9 positives at t1. In the discordant subgroup, this was even more pronounced (60.0%) than in the concordant subgroup (37.0%). Therefore, the combination of the GP and PHQ-9 assessments may be associated with a decreased likelihood of false-positives.

The accurate ruling-out of depression is also of great importance. In our analysis, we found that patients who were not rated as depressive by their GP had less PHQ-9 positive results at t1 and t2. Other studies have already shown that GPs are good at ruling-out depression [3, 21, 22]. PHQ-9 results were more reliable in the concordant subgroup, in particular when PHQ-9 scores were <5 at t1 and t2. This suggests that a better ruling-out of a depression is achieved when the GP’s rating is combined with negative PHQ-9 results.

In contrast to that, patients with intermediate PHQ-9 scores (5–10) remain in a “grey area” which seems to represent a major challenge for diagnostic decision making, especially if GPs are less confident in their abilities to identify depression [24]. However, it is of great importance to increase reliable diagnostic decision making in cases with milder forms of depression which are characterized by high diagnostic uncertainty.

In the discordant subgroup, patients’ PHQ-9 scores were more often just above or just below the cut-off at t1, so they were more likely to drift above or below the cut-off at t2. Recognizing stable and clear cases with pronounced depressive symptoms or without symptoms seems to be easier for GPs, while diagnosing patients with subthreshold symptoms is a major challenge. GPs are more likely to identify a more severe depression indicated by higher PHQ-9 results and are more likely to exclude a depression diagnosis correctly if the PHQ-9 result is low.

Furthermore, it has to be taken into account that among 20 patients with PHQ-9 positive results at t1 in the discordant subgroup, a significant proportion (40.0%) of patients received a positive result at t2 again, which implies the general tendency of chronicity [41, 42]. Nevertheless, there is a remarkable difference to the concordant subgroup where chronicity rates are higher (63.0%). This might indicate again, that a combination of GP heuristics and screening questionnaire could improve the diagnostic process of patients with severe depression and a high risk of chronicity, which needs to be investigated in further studies.

### Limitations

Our analysis has several limitations. First, influential life circumstances, such as therapy consultation, medication or critical life events were not measured between t1 and t2. This could have an impact on the interpretation of the results as we do not know what happened in the meantime between t1 and t2. Future studies should address this point to analyze the impact of such factors. Secondly, the results were derived by a secondary analysis. Therefore, the findings of the present study should be validated within further diagnostic studies. Thirdly, the GP rating of the patient as depressive is a subjective evaluation based on implicit GP heuristics which were used to indicate a depression diagnosis (yes/no). However, we did not identify which specific GP heuristics were used to diagnose depression. Therefore, an in-depth exploration of GP heuristics and the differentiation from psychiatric diagnostics seems to be of great value. Moreover, we could not verify whether the GP explicitly used the diagnostic criteria of depression during patient assessment. Further limitations arise from the patients who refused to participate in the study at t2. These non-responders were on average younger than responders, which might have an effect on the appearance of depression diagnoses at t2. An important limitation is given by the PHQ-9 itself. The questionnaire is well suited as a screening method for depression, but it cannot be used to obtain a reliable and definite psychological diagnosis. To be certain, such a diagnosis must be confirmed by a standardized diagnostic interview, which was not performed in this study. As it is difficult to determine the accurate proportion of false-positives and false-negatives without a reference standard, further studies need to investigate the accuracy of GP diagnoses in combination with screening questionnaires compared to standardized diagnostic interviews as a reference standard.

## Conclusion

The combination of the PHQ-9 and the GP rating might improve diagnostic decision making regarding depression in general practice. Therefore, it is necessary to combine common diagnostic methods and GP heuristics to improve the positive predictive value of screening questionnaires like the PHQ-9. Thus, a questionnaire which specifically considers the GP heuristics as well as the psychiatric criteria might be useful. Further studies are necessary to identify explicit GP heuristics which might increase diagnostic accuracy in primary care.

## References

[1] Demyttenaere K, Bruffaerts R, Posada-Villa J, Gasquet I, Kovess V, Lepine JP, et al. Prevalence, severity, and unmet need for treatment of mental disorders in the World Health Organization World Mental Health Surveys. JAMA. 2004;291(21):2581–90. doi: 10.1001/jama.291.21.2581 15173149

[2] Jacobi F, Höfler M, Siegert J, Mack S, Gerschler A, Scholl L, et al. Twelve-month prevalence, comorbidity and correlates of mental disorders in Germany: the Mental Health Module of the German Health Interview and Examination Survey for Adults (DEGS1-MH). Int J Methods Psychiatr Res. 2014;23(3):304–19. doi: 10.1002/mpr.1439 24729411 PMC6878234

[3] Mitchell AJ, Vaze A, Rao S. Clinical diagnosis of depression in primary care: a meta-analysis. Lancet (London, England). 2009;374(9690):609–19. 10.1016/S0140-6736(09)60879-5 19640579

[4] Mitchell AJ, Rao S, Vaze A. Can general practitioners identify people with distress and mild depression? A meta-analysis of clinical accuracy. Journal of Affective Disorders. 2011;130(1):26–36. doi: 10.1016/j.jad.2010.07.028 20708274

[5] Mitchell AJ, Rao S, Vaze A. International comparison of clinicians’ ability to identify depression in primary care: meta-analysis and meta-regression of predictors. British Journal of General Practice. 2011;61(583):e72–e80. doi: 10.3399/bjgp11X556227 21276327 PMC3026173

[6] Kruse J, Schmitz N, Wöller W, Heckrath C, Tress W. [Why does the general practitioner overlooks psychological disorders in his patient?]. Psychother Psychosom Med Psychol. 2004;54(2):45–51. 14872390 10.1055/s-2003-814791

[7] Schneider A, Wartner E, Schumann I, Hörlein E, Henningsen P, Linde K. The impact of psychosomatic co-morbidity on discordance with respect to reasons for encounter in general practice. Journal of psychosomatic research. 2013;74(1):82–5. doi: 10.1016/j.jpsychores.2012.09.007 23272993

[8] Bühring P. 121. Deutscher Ärztetag in Erfurt: Mehr Aufmerksamkeit für psychische Erkrankungen. Dtsch Arztebl International. 2018;115(17):812–4. German.

[9] Schäfer I, Hansen H, Schön G, Höfels S, Altiner A, Dahlhaus A, et al. The influence of age, gender and socio-economic status on multimorbidity patterns in primary care. First results from the multicare cohort study. BMC health services research. 2012;12:89. doi: 10.1186/1472-6963-12-89 22471952 PMC3348059

[10] Haddad M. Depression in adults with a chronic physical health problem: treatment and management. International journal of nursing studies. 2009;46(11):1411–4. doi: 10.1016/j.ijnurstu.2009.08.007 19748379

[11] Craven MA, Bland R. Depression in primary care: current and future challenges. Canadian journal of psychiatry Revue canadienne de psychiatrie. 2013;58(8):442–8. doi: 10.1177/070674371305800802 23972105

[12] Fink P. Surgery and medical treatment in persistent somatizing patients. Journal of psychosomatic research. 1992;36(5):439–47. doi: 10.1016/0022-3999(92)90004-l 1535658

[13] Holmes A, Christelis N, Arnold C. Depression and chronic pain. The Medical journal of Australia. 2013;199(6):S17–S20. doi: 10.5694/mja12.10589 25370278

[14] Davidsen AS, Fosgerau CF. What is depression? Psychiatrists’ and GPs’ experiences of diagnosis and the diagnostic process. International journal of qualitative studies on health and well-being. 2014;9:24866. doi: 10.3402/qhw.v9.24866 25381757 PMC4224702

[15] Dilling H, Freyberger HJ. Taschenführer zur ICD-10-Klassifikation psychischer Störungen: nach dem Pocket Guide von J.E. Cooper. 9th ed. Bern; 2019.

[16] Wockenfuss R, Frese T, Herrmann K, Claussnitzer M, Sandholzer H. Three-and four-digit ICD-10 is not a reliable classification system in primary care. Scandinavian journal of primary health care. 2009;27(3):131–6. doi: 10.1080/02813430903072215 19565412 PMC3413183

[17] Lucassen P, van Rijswijk E, van Weel-Baumgarten E, Dowrick C. Making fewer depression diagnoses: beneficial for patients? Mental health in family medicine. 2008;5(3):161–5. 22477864 PMC2777570

[18] Kessler RC, Berglund P, Demler O, Jin R, Koretz D, Merikangas KR, et al. The epidemiology of major depressive disorder: results from the National Comorbidity Survey Replication (NCS-R). Jama. 2003;289(23):3095–105. doi: 10.1001/jama.289.23.3095 12813115

[19] Pedersen SH, Stage KB, Bertelsen A, Grinsted P, Kragh-Sørensen P, Sørensen T. ICD-10 criteria for depression in general practice. J Affect Disord. 2001;65(2):191–4. doi: 10.1016/s0165-0327(00)00268-8 11356244

[20] Gunn J, Elliott P, Densley K, Middleton A, Ambresin G, Dowrick C, et al. A trajectory-based approach to understand the factors associated with persistent depressive symptoms in primary care. J Affect Disord. 2013;148(2–3):338–46. doi: 10.1016/j.jad.2012.12.021 23375580

[21] Fernández A, Pinto-Meza A, Bellón JA, Roura-Poch P, Haro JM, Autonell J, et al. Is major depression adequately diagnosed and treated by general practitioners? Results from an epidemiological study. General hospital psychiatry. 2010;32(2):201–9. doi: 10.1016/j.genhosppsych.2009.11.015 20302995

[22] Carey M, Jones K, Meadows G, Sanson-Fisher R, D’Este C, Inder K, et al. Accuracy of general practitioner unassisted detection of depression. Aust N Z J Psychiatry. 2014;48(6):571–8. doi: 10.1177/0004867413520047 24413807 PMC4230951

[23] Menear M, Doré I, Cloutier A-M, Perrier L, Roberge P, Duhoux A, et al. The influence of comorbid chronic physical conditions on depression recognition in primary care: a systematic review. Journal of psychosomatic research. 2015;78(4):304–13. doi: 10.1016/j.jpsychores.2014.11.016 25676334

[24] Sinnema H, Terluin B, Volker D, Wensing M, Van Balkom A. Factors contributing to the recognition of anxiety and depression in general practice. BMC family practice. 2018;19(1):1–10. 29935537 10.1186/s12875-018-0784-8 PMC6015659

[25] Schumann I, Schneider A, Kantert C, Löwe B, Linde K. Physicians’ attitudes, diagnostic process and barriers regarding depression diagnosis in primary care: a systematic review of qualitative studies. Family practice. 2012;29(3):255–63. doi: 10.1093/fampra/cmr092 22016322

[26] Schneider A, Mayer V, Dinkel A, Wagenpfeil S, Linde K, Henningsen P. Educational intervention to improve diagnostic accuracy regarding psychological morbidity in general practice. Zeitschrift für Evidenz, Fortbildung und Qualität im Gesundheitswesen. 2019;147:20–7. 31623979 10.1016/j.zefq.2019.08.006

[27] Pilars de Pilar M, Abholz H-H, Becker N, Sielk M. How do general practitioners deal with patients they do not consider to be depressed but who are classified as such according the PHQ-9? Psychiatrische Praxis. 2011;39(2):71–8. 10.1055/s-0031-1276897 21969231

[28] Levis B, Sun Y, He C, Wu Y, Krishnan A, Bhandari PM, et al. Accuracy of the PHQ-2 Alone and in Combination With the PHQ-9 for Screening to Detect Major Depression: Systematic Review and Meta-analysis. JAMA. 2020;323(22):2290–300. doi: 10.1001/jama.2020.6504 32515813 PMC7284301

[29] Trivedi MH. Major Depressive Disorder in Primary Care: Strategies for Identification. The Journal of clinical psychiatry. 2020;81(2). doi: 10.4088/JCP.UT17042BR1C 32220155

[30] Thombs BD, Kwakkenbos L, Levis AW, Benedetti A. Addressing overestimation of the prevalence of depression based on self-report screening questionnaires. Canadian Medical Association Journal. 2018;190(2):E44–E9. doi: 10.1503/cmaj.170691 29335262 PMC5770251

[31] Gilbody S, Richards D, Brealey S, Hewitt C. Screening for depression in medical settings with the Patient Health Questionnaire (PHQ): a diagnostic meta-analysis. Journal of general internal medicine. 2007;22(11):1596–602. doi: 10.1007/s11606-007-0333-y 17874169 PMC2219806

[32] Spangenberg L, Forkmann T, Braehler E, Glaesmer H. The association of depression and multimorbidity in the elderly: implications for the assessment of depression. Psychogeriatrics. 2011;11(4):227–34. doi: 10.1111/j.1479-8301.2011.00375.x 22151242

[33] Levis B, Benedetti A, Thombs BD. Accuracy of Patient Health Questionnaire-9 (PHQ-9) for screening to detect major depression: individual participant data meta-analysis. BMJ. 2019;365:l1476. doi: 10.1136/bmj.l1476 30967483 PMC6454318

[34] Joffres M, Jaramillo A, Dickinson J, Lewin G, Pottie K, Shaw E, et al. Recommendations on screening for depression in adults. Cmaj. 2013;185(9):775–82. doi: 10.1503/cmaj.130403 23670157 PMC3680556

[35] Mitchell AJ, Yadegarfar M, Gill J, Stubbs B. Case finding and screening clinical utility of the Patient Health Questionnaire (PHQ-9 and PHQ-2) for depression in primary care: a diagnostic meta-analysis of 40 studies. BJPsych open. 2016;2(2):127–38. doi: 10.1192/bjpo.bp.115.001685 27703765 PMC4995584

[36] Thombs BD, Coyne JC, Cuijpers P, de Jonge P, Gilbody S, Ioannidis JP, et al. Rethinking recommendations for screening for depression in primary care. Cmaj. 2012;184(4):413–8. doi: 10.1503/cmaj.111035 21930744 PMC3291670

[37] National Collaborating Center for Mental Health The NICE guideline on the management and treatment of depression in adults (updated edition). London (UK): National Institute for Health and Clinical Excellence; 2010.

[38] Siu AL, Force atUPST. Screening for Depression in Adults: US Preventive Services Task Force Recommendation Statement. JAMA. 2016;315(4):380–7. 26813211 10.1001/jama.2015.18392

[39] Kroenke K, Spitzer RL, Williams JB. The PHQ-9: validity of a brief depression severity measure. J Gen Intern Med. 2001 Sep;16(9):606–13. doi: 10.1046/j.1525-1497.2001.016009606.x ; PMCID: PMC1495268. 11556941 PMC1495268

[40] Costantini L, Pasquarella C, Odone A, Colucci ME, Costanza A, Serafini G, et al. Screening for depression in primary care with Patient Health Questionnaire-9 (PHQ-9): A systematic review. Journal of Affective Disorders. 2021;279:473–83. doi: 10.1016/j.jad.2020.09.131 33126078

[41] Verhoeven J, Verduijn J, Milaneschi Y, Beekman A, Penninx B. The Clinical Course of Depression: Chronicity is the Rule Rather than the Exception. European Psychiatry. 2017;41(S1):S144–S5.

[42] Ten Have M, de Graaf R, van Dorsselaer S, Tuithof M, Kleinjan M, Penninx B. Recurrence and chronicity of major depressive disorder and their risk indicators in a population cohort. Acta psychiatrica Scandinavica. 2018;137(6):503–15. doi: 10.1111/acps.12874 29577236

