# Supplementary material for: Combining the GP’s assessment and the PHQ-9 questionnaire leads to more reliable and clinically relevant diagnoses in primary care
Source: PLoS One. 2022 Oct 21;17(10):e0276534. doi: 10.1371/journal.pone.0276534 (PMC9586376; doi:10.1371/journal.pone.0276534)
Supplement: Attachment — Submitted filename: 04 PHQ9 vs. GP dep, PLoS (Feb).docx [file pone.0276534.s001.docx]

This is a useful study examining concordance between a depression measure (PHQ-9) and general practitioners’ (GP) ratings of depression. Strengths include sample size (n=364), longitudinal assessment at two time points (baseline and 3 month follow-up) and generally appropriate analyses.

Comments

1. The greater reliability of the concordant group could be partly due to the possibility that those with GP depression at t1 had higher PHQ-9 scores and those with GP no depression had lower PHQ-9 scores, compared to the dtiscordant group. Thus, if the discordant group had PHQ-9 scores closer to just above or just below 10 at t1, they would be more likely to drift above or below 10 at t2. This could be examined by showing mean PHQ-9 scores in Table 2. An example is shown below.

|  | **Concordant subgroup at t1 (N=208)** | | | |
| --- | --- | --- | --- | --- |
|  | Depression  at t2 | Mean (SD) PHQ-9 | No depression at t2 | Mean (SD) PHQ-9 |
| Depression at t1 | 17 |  | 10 |  |
| No depression at t1 | 15 |  | 166 |  |
|  | **Discordant subgroup at t1 (N=55)** | | | |
|  | Depression  at t2 | Mean (SD) PHQ-9 | No depression at t2 | Mean (SD) PHQ-9 |
| Depression at t1 | 8 |  | 12 |  |
| No depression at t1 | 7 |  | 28 |  |

2. Table 1.
   a. Define what PHQ-9 Cutoff value is, either in the column heading itself or a footnote.
   b. Except for the Total column, the percentages represent row rather than column percentages. This is different from what published tables usually show and does not allow direct comparison of the “Yes” and “No” groups. For example, the proportion of women who were Yes and No for depression rating by the GP were 55/85 and 142/271, or 64.7% and 52.4%, respectively. This latter numbers directly show the proportional difference in women between the depressed and nondepressed group. The current row percentages (27.1% and 70.1%) do not show this. Thus, the authors should change percentages in table to reflect column rather than row percentages.
3. Were GPs blinded to PHQ-9 when they made their assessment of depression? If not (or if we don’t know) this should be added as a study limitation in the Discussion, since knowledge of the PHQ-9 could have influenced their assessment of depression (i.e., the two methods would not be entirely independent).
4. Lines 81-85 are overstated:
   a. The sentence “Thus, a PHQ-9 positive result can only give a hint towards a possible depression and needs to be confirmed by a structured diagnostic interview.” There is a large amount of data supporting the construct validity of scores of 10 or greater representing clinically significant depressive symptoms. Sometimes too much is made of a “major depressive disorder diagnosis”. I might rephrase the sentence to something like: “Whereas a PHQ-9 score ≥10 may represent clinically significant depressive symptoms, a structured diagnostic interview is needed to confirm the presence of major depressive disorder.”
   b. The sentence: “Therefore, the standardised and legitimised use of screening questionnaires in primary care has not yet been established.” This is an overstatement. Canadian and some European guidelines are less enthusiastic about depression screening than US guidelines and this nuance should be reflected rather than just stating a “legitimized use of screening questionnaires in primary care has not yet been established.”
5. Line 123 states: “A score of 10 thus indicates the presence of depression [32].” This is too simplistic. Instead, a score of 10 or greater represents a moderate level of depressive symptoms. The way the authors state it sounds like a “depression diagnosis.” Also, the reference for a cutpoint of 10 is not reference 32 (Spitzer 1999) but instead the Kroenke et al 2001 reference on the PHQ-9 in J Gen Intern Med.
6. Line 145 – A few sentences with more detail describing the “training intervention” would be helpful since it could have affected GP diagnosis rates.
7. Lines 183-185 – Could the authors explain a little better “Limits of Agreement” – is this similar or different from 95% CI. The interpretation of Bland-Altman graphs will be unfamiliar to many readers
8. Minor points
   a. Line 54. “Subliminal” should probably be “subthreshold”
   b. Bland-Altman plots text/font, along axes, is difficult to read, and a more production-ready version of these graphs should be provided.
